# Validation of the novel Eosinophilic Esophagitis Impact Questionnaire

**DOI:** 10.1186/s41687-023-00654-z

**Published:** 2023-11-27

**Authors:** Eilish McCann, Mirna Chehade, Jonathan M. Spergel, Andrew Yaworsky, Tara Symonds, Jonathan Stokes, Sarette T. Tilton, Xian Sun, Siddhesh Kamat

**Affiliations:** 1 Regeneron Pharmaceuticals, Tarrytown, NY USA; 2 Mount Sinai Center for Eosinophilic Disorders, Icahn School of Medicine at Mount Sinai, New York, NY USA; 3 Division of Allergy and Immunology, Department of Pediatrics, Children’s Hospital of Philadelphia, Perelman School of Medicine at University of Pennsylvania School of Medicine, Philadelphia, PA USA; 4 Adelphi Values, Boston, MA USA; 5 Clinical Outcomes Solutions, Folkestone, Kent, CT19 4RH UK; 6 Adelphi Values, Boston, MA USA; 7 Sanofi Global, Bridgewater, NJ USA

## Abstract

**Background:**

Eosinophilic esophagitis (EoE) has a detrimental effect on health-related quality of life (HRQOL). The Eosinophilic Esophagitis Impact Questionnaire (EoE-IQ) is a novel patient-reported outcome (PRO) measure assessing the impact of EoE on HRQOL. To assess suitability of the EoE-IQ, its measurement properties were evaluated.

**Methods:**

Using baseline and week 24 data from the pivotal, randomized, placebo-controlled, multinational phase 3 R668-EE-1774 trial (NCT03633617) of dupilumab, we evaluated EoE-IQ’s measurement properties (including reliability, construct and known-groups validity, and ability to detect change) and established the threshold for change in scores that can be considered clinically meaningful.

**Results:**

The analysis population comprised 239 adults and adolescents with EoE. Mean age was 28.1 (standard deviation, 13.14) years; 63.6% were male, and 90.4% were White. Reliability estimates for the EoE-IQ average score exceeded acceptable thresholds for patients who were stable as indicated by ratings of Patient Global Impression of Severity (PGIS) and Change (PGIC) (intraclass correlation coefficients, 0.75 and 0.81). Construct validity correlations with other EoE-specific PRO scores were moderate at baseline (|r|= 0.44–0.60) and moderate to strong at week 24 (|r|= 0.61–0.72). In known-groups analysis, EoE-IQ average score discriminated among groups of patients at varying EoE severity levels defined by PGIS scores. A ≥ 0.6-point reduction in EoE-IQ average score (where scores range from 1 to 5, with higher scores indicating worse HRQOL) from baseline to week 24 can be considered clinically meaningful.

**Conclusions:**

The EoE-IQ’s measurement properties are acceptable, making it a valid, reliable measure of the HRQOL impacts of EoE among adults and adolescents.

*Trial registration*: ClinicalTrials.gov, NCT03633617. Registered August 14, 2018, https://clinicaltrials.gov/study/NCT03633617.

**Supplementary Information:**

The online version contains supplementary material available at 10.1186/s41687-023-00654-z.

## Background

Eosinophilic esophagitis (EoE) is a chronic, progressive, type 2 inflammatory disease that has a substantial impact on health-related quality of life (HRQOL) and that is increasing in incidence [1–5]. Diagnosis of EoE is based on symptoms and esophageal eosinophilic infiltration at 15 or more eosinophils per high-power field (eos/hpf) [1]. Symptoms of EoE may appear in childhood or adulthood and include abdominal pain, heartburn, regurgitation, and vomiting. The most common symptom among adolescents and adults, however, is dysphagia, which is associated with esophageal food impactions that may require urgent endoscopic removal. The symptoms of EoE can result in negative impacts to social and emotional well-being, sleep impairments, decreased work or school productivity, and increased healthcare resource utilization [2, 6–10].

It is important to understand the impact of EoE symptoms on HRQOL when evaluating new therapeutics. The United States Food and Drug Administration recommends the use of patient-reported outcome (PRO) measures in clinical trials as a way to measure the impact of treatment on symptoms and HRQOL [11]. Although existing PRO measures are available to assess the impact of EoE and other esophageal conditions on HRQOL [12–14], none were developed for use in both adult and adolescent EoE populations in a clinical trial setting. Therefore, the novel 11-item Eosinophilic Esophagitis Impact Questionnaire (EoE-IQ) was developed to assess various aspects of HRQOL in adults and adolescents with EoE.

The initial development of the EoE-IQ was informed by a literature review and advice meetings with EoE experts. The findings of this research led to the selection of 11 HRQOL-related impact concepts for inclusion in the draft questionnaire. An initial 11-item draft of the EoE-IQ was tested in a set of 23 cognitive debriefing interviews. These interviews were designed to assess patients’ ability to understand the content of the EoE-IQ and assess the relevance and comprehensiveness of the concepts measured by the questionnaire. During 11 of the interviews, the EoE-IQ was debriefed in full with a combination of adult (n = 5) and adolescent (n = 6) participants, while the remaining 12 interviews were conducted with adult participants who were asked to read the EoE-IQ and provide high-level feedback on the questionnaire. Findings from these interviews indicated that the EoE-IQ was well understood and comprehensively measured concepts relevant to the experience of patients with EoE (Additional file 1: Section 1).

Subsequent to these interviews, the EoE-IQ was implemented in a randomized, placebo-controlled trial investigating the efficacy and safety of dupilumab for adult and adolescent patients with EoE (R668-EE-1774; NCT03633617) [15].

Before a new PRO can be considered valid and reliable for use in a target population, it is important to assess its measurement properties, including reliability, construct validity, and responsiveness. The aim of this analysis was to evaluate the validity of the EoE-IQ for measuring HRQOL in adults and adolescents with EoE in a clinical trial. The objectives were to provide evidence of the measurement properties of the EoE-IQ and to determine a threshold for meaningful change using questionnaire responses gathered in the R668-EE-1774 study.

## Methods

### Study design and analysis population

R668-EE-1774 (NCT03633617) was a phase 3, randomized, double-blind, placebo-controlled clinical trial to investigate the efficacy and safety of dupilumab in adult and adolescent patients (aged ≥ 12 years) with EoE [15]. Co-primary endpoints in R668-EE-1774 were (1) the proportion of patients achieving peak esophageal intraepithelial eosinophil count of ≤ 6 eos/hpf at week 24 (i.e., a count of eosinophils in a esophageal biopsy that indicates histological disease remission) and (2) absolute change in Dysphagia Symptom 202Questionnaire (DSQ) biweekly total score from baseline to 24 weeks. Absolute change in EoE-IQ average score from baseline to week 24 was a secondary endpoint in R668-EE-1774.

The study comprised 3 main parts and a follow-up period: Part A (N = 81) and Part B (N = 240), each consisting of a 24-week double-blind treatment period; Part C, a 28-week extended active treatment period (in which Part B patients remained blinded to current treatment regimen and both Part A and Part B patients remained blinded to prior treatment allocation); and a 12-week follow-up period after the end of the extended active treatment period for participants who entered into Part C or following the end of Parts A or B for participants who did not enter Part C. Given the larger sample size in Part B relative to Part A, the primary psychometric analyses reported here were conducted using EoE-IQ data from Part B; analyses conducted with Part A data yielded similar findings (data not shown). Baseline characteristics are presented for the PRO analysis population, which included all randomized patients in Part B who completed at least 1 of 3 PRO measures administered at baseline in the trial: the EoE-IQ; the DSQ, which evaluated dysphagia symptoms; or the EoE Symptom Questionnaire (EoE-SQ), which evaluated EoE symptoms other than dysphagia (N = 239 for Part B). Absolute changes in frequency and severity of symptoms evaluated by the EoE-SQ from baseline to week 24 were secondary endpoints in R668-EE-1774. For all psychometric analyses, no imputation of missing responses was performed; only observed responses were included.

### Overview of the EoE-IQ

The EoE-IQ contains 11 items that evaluate the impact of EoE on patients’ lives in relation to emotional functioning, social impact, school/work impact, and sleep disruption using a 7-day recall period. Items are scored using a 5-point verbal rating scale (1 = Not at all [impacted]; 2 = A little; 3 = Somewhat; 4 = Quite a bit; 5 = Extremely [impacted]). The EoE-IQ average score is computed as the sum of the scores from nonmissing responses divided by the number of items with a nonmissing response. The average score can range from 1 to 5, with a higher score indicating worse HRQOL. Study participants completed the EoE-IQ electronically at scheduled site visits at baseline, week 12, and week 24 during the double-blind treatment period.

### Supporting measures used to validate the EoE-IQ

Four EoE-related PRO measures were used in the psychometric evaluation of the EoE-IQ: the Patient Global Impression of Severity (PGIS), the Patient Global Impression of Change (PGIC), the EoE-SQ, and the DSQ (Additional file 1: Table S1).

The PGIS is a single-item questionnaire evaluating patients’ difficulty swallowing food over the past week on a 4-point scale (0 = None to 3 = Severe), with lower scores indicating lower symptom severity. The PGIC is a single-item questionnaire evaluating patients’ overall change in difficulty swallowing food since beginning study medication on a 7-point scale (0 = Very much better to 6 = Very much worse), with lower scores indicating greater improvement in difficulty swallowing food.

The EoE-SQ measures frequency and severity of EoE symptoms other than dysphagia and pain with swallowing during the past 7 days: chest pain, stomach pain, burning feeling in the chest, food or liquid coming back up into the throat, and throwing up. Frequency is rated on a 5-point scale (1 = Never to 5 = More than once a day) and severity on an 11-point numeric rating scale (0 = No symptom to 10 = Worst possible symptom) for each symptom. EoE-SQ Frequency scores are calculated as the sum of responses to 5 items and thus range from 5 to 25, with higher scores indicating higher frequency of symptoms. EoE-SQ Severity scores are calculated as the sum of the responses to 3 items (chest pain, stomach pain, and burning feeling in the chest) and thus range from 0 to 30, with higher scores indicating more severe symptoms.

The DSQ is a 4-item questionnaire evaluating the daily frequency and severity of dysphagia associated with EoE [16, 17]. The first question of the DSQ asks if the patient ate solid food that day; if they answer “yes,” they are then asked Questions 2 (frequency) and 3 (severity), which are used in the scoring of the DSQ. A fourth DSQ item, which assesses pain when swallowing food, was also administered but did not contribute to the DSQ scoring in this analysis. The DSQ biweekly total score is calculated over a 14-day period (as per the developer instructions) and ranges from 0 to 84, with higher scores indicating worse dysphagia burden.

Clinical measures assessed in R668-EE-1774 included the peak esophageal intraepithelial eosinophil count and the Eosinophilic Esophagitis Endoscopic Reference Score (EoE-EREFS) (Additional file 1: Table S1). Peak esophageal intraepithelial eosinophil count is the maximum quantity of eosinophils in the most inflamed high-power fields across 3 regions of the esophagus and can be categorized into 3 levels: ≤ 6 eos/hpf (histological remission); > 6 to < 15 eos/hpf; and ≥ 15 eos/hpf (threshold for EoE diagnosis and inclusion in R668-EE-1774). The EoE-EREFS is a system for scoring inflammatory and remodeling features of disease for the proximal and distal esophageal regions [18]; the total score (summing scores for the 2 regions) ranges from 0 to 18, with higher scores indicating greater disease activity*.*

### Statistical analyses

#### Score distribution

Descriptive statistics were summarized for the EoE-IQ score at baseline and week 24 and for the corresponding change in scores from baseline. Floor (worst outcome) or ceiling (best outcome) effects were defined as > 20% of patients with the worst score (5) or the best score (1) at baseline, respectively. Inter-item and corrected item-total correlations (which were computed without the corresponding item) were determined in order to evaluate potential redundancy and assess the strength of the relationships of the items. Correlations > 0.30 and < 0.80 are generally considered appropriate.

#### Reliability

To evaluate internal consistency, or the extent to which EoE-IQ items measure the same concept, associations between items and the overall scale were assessed through Cronbach’s coefficient alpha. Cronbach’s coefficient alphas ≥ 0.70 indicated acceptable internal consistency [19]. To evaluate test-test reliability, or the reproducibility of EoE-IQ average scores over time among stable patients under the same assessment conditions, intraclass correlation coefficients (ICCs) were computed using 2-way, mixed-effect analysis of variance (ANOVA) with absolute agreement for single measures [20] between week 12 (test) and week 24 (retest). Stable patient subsets were defined as patients with the same PGIS score at test and retest and patients with the same PGIC score at test and retest. An ICC of ≥ 0.70 indicated acceptable reliability [19, 21].

#### Construct validity

Pearson correlations between EoE-IQ average scores and scores on the supporting measures at baseline and week 24 were determined to assess convergent and divergent validity. Correlations of < 0.3 were considered to be weak or small, ≥ 0.3 to < 0.7 to be moderate, ≥ 0.7 to < 0.9 to be strong, and ≥ 0.9 to be very strong [22, 23]. Specific hypotheses for the anticipated correlation between the EoE-IQ average score and other measures were as follows: (1) moderate to strong positive correlation with the PGIS score; (2) moderate positive correlations with the EoE-SQ and DSQ scores; (3) small to moderate positive correlation with the peak and categorized peak esophageal intraepithelial eosinophil count (assessed only at week 24, as all patients had ≥ 15 eos/hpf at baseline per trial inclusion criteria); and (4) small positive correlation with the EoE-EREFS. An ANOVA/t-test was used to compare categories of PGIS at baseline and week 24 and peak eosinophil count at week 24 to determine known-groups validity.

#### Responsiveness

To evaluate the extent to which the EoE-IQ detects change, correlations were calculated between change in EoE-IQ average score from baseline to week 24 and changes as assessed by the PGIS, PGIC, and other supporting PRO and clinical measures (DSQ, EoE-SQ, EoE-EREFS, and peak and categorized peak esophageal intraepithelial eosinophil count).

ANOVAs were performed for differences in change in average EoE-IQ score from baseline to week 24 by responsiveness groups defined by (1) PGIS changes (improved: ≥ 1-point improvement; no change: 0-point change; worsened: ≥ 1-point worsening), (2) PGIC score (better: “A little better,” “Moderately better,” “Very much better”; no change: “No change”; worse: “A little worse,” “Moderately worse,” “Very much worse”), and (3) peak esophageal intraepithelial eosinophil count (responder: ≤ 6 eos/hpf; nonresponder: > 6 eos/hpf).

In addition, standardized effect size (SES) statistics were calculated for within- and between-group changes. An SES of 0.20 to < 0.50 is considered to be small, 0.50 to < 0.80 to be moderate, and ≥ 0.80 to be large [22].

#### Interpretation of change

To explore meaningful within-patient change in EoE-IQ average scores, anchor-based analyses were performed using the PGIS and PGIC as potential anchor measures. Supportive distribution-based statistics were also computed. Median and mean change in EoE-IQ average scores were computed with 95% confidence intervals (CIs) by category of PGIS change from baseline to week 24 and PGIC score at week 24. A responsiveness correlation of |r|≥ 0.371 (based on the conversion from an effect size of 0.8, representing a large effect [24]) was required for the potential anchor measures to be considered adequately related to the EoE-IQ average score. Key anchor levels were represented by a PGIS change of − 1 (i.e., 1-point improvement) and a PGIC score of “A little better.” Empirical cumulative distribution function (CDF) plots were generated by anchor levels to provide visual support for the meaningful within-patient change threshold estimates. In addition, supportive distribution-based estimates of change were computed as the half standard deviation (SD) of baseline scores, and standard error of measurement (SEM) was computed using the test–retest ICC as a reliability estimate.

## Results

### Patient demographics and clinical characteristics

The Part B PRO analysis population comprised 239 patients: 79 adolescents aged 12 to < 18 years and 160 adults aged 18 years or older (Table 1). The overall sample had a mean age of 28.1 years (SD, 13.14; range, 12–68); 63.6% were male, and 90.4% were White. The most common atopic comorbidity was allergic rhinitis (64.0%), followed by asthma (44.8%) and atopic dermatitis (25.9%).

**Table 1:** Patient demographic and disease characteristics at baseline (patient-reported outcome population analysis set: part B)

Characteristic	Adolescents aged 12 to < 18 years (n = 79)	Adults aged ≥ 18 years (n = 160)	Overall PRO population analysis sample (N = 239)
*Age* (years)			
Mean (SD)	15.0 (1.62)	34.7 (11.32)	28.1 (13.14)
Median	15.0	35.0	24.0
Q1: Q3	14.0: 16.0	24.0: 41.5	16.0: 38.0
Min: max	12: 17	18: 68	12: 68
*Sex, n* (%)			
Male	57 (72.2%)	95 (59.4%)	152 (63.6%)
Female	22 (27.8%)	65 (40.6%)	87 (36.4%)
*Race, n* (%)			
White	64 (81.0%)	152 (95.0%)	216 (90.4%)
Black or African American	7 (8.9%)	1 (0.6%)	8 (3.3%)
Asian	2 (2.5%)	3 (1.9%)	5 (2.1%)
Other	6 (7.6%)	1 (0.6%)	7 (2.9%)
Not reported	0	3 (1.9%)	3 (1.3%)
*Ethnicity, n* (%)			
Not Hispanic or Latino	76 (96.2%)	149 (93.1%)	225 (94.1%)
Hispanic or Latino	3 (3.8%)	10 (6.3%)	13 (5.4%)
Unknown	0	1 (0.6%)	1 (0.4%)
*Atopic comorbidity, n* (%)			
Allergic rhinitis	57 (72.2%)	96 (60.0%)	153 (64.0%)
Asthma	33 (41.8%)	29 (18.1%)	107 (44.8%)
Atopic dermatitis	45 (57.0%)	62 (38.8%)	62 (25.9%)

PRO, patient-reported outcome; Q1, quartile 1; Q3, quartile 3; SD, standard deviation

### Score distribution

In Part B, of the 239 patients in the PRO population, 224 had an EoE-IQ score at baseline and 212 had an EoE-IQ score at week 24 (Table 2). As all assessments were administered electronically and did not allow for individual items to be skipped, there were no item-level missing data. Mean baseline EoE-IQ average score was 2.27 (SD, 0.77) (Table 3). The EoE-IQ items with the highest mean baseline scores, reflecting the greatest negative impact on HRQOL, were Item 1 (Bothered by symptoms: 3.3), Item 2 (Worried about swallowing: 3.1), and Item 5 (Worried about swallowing in public: 2.9). There were no floor or ceiling effects, thus allowing the potential to show both improvement and worsening in HRQOL impact over time. EoE-IQ scores decreased (indicating improvement) during the study to a mean of 1.57 (SD, 0.65) at week 24.

**Table 2:** Summary of EoE-IQ item-level response distribution at baseline and week 24

Time point/EoE-IQ item	Mean item score (SD)	95% CI	Frequency (%)				
			Not at all	A little	Somewhat	Quite a bit	Extremely
*Baseline EoE-IQ score* (*n* = 224)							
1. Bothered by symptoms	3.3 (0.89)	3.22–3.45	1 (0.4)	41 (18.3)	84 (37.5)	78 (34.8)	20 (8.9)
2. Worried about swallowing	3.1 (1.09)	2.98–3.27	14 (6.3)	53 (23.7)	73 (32.6)	59 (26.3)	25 (11.2)
3. Worried about choking	2.6 (1.29)	2.47–2.81	52 (23.2)	65 (29.0)	39 (17.4)	48 (21.4)	20 (8.9)
4. Embarrassed	2.0 (1.14)	1.90–2.20	95 (42.4)	60 (26.8)	40 (17.9)	21 (9.4)	8 (3.6)
5. Worried about swallowing in public	2.9 (1.27)	2.77–3.10	39 (17.4)	39 (17.4)	73 (32.6)	43 (19.2)	30 (13.4)
6. Difficulty in social activities	2.3 (1.23)	2.19–2.51	71 (31.7)	60 (26.8)	53 (23.7)	24 (10.7)	16 (7.1)
7. Family relationships	1.6 (1.01)	1.46–1.73	151 (67.4)	33 (14.7)	26 (11.6)	8 (3.6)	6 (2.7)
8. Friendships	1.6 (1.01)	1.50–1.77	141 (62.9)	46 (20.5)	20 (8.9)	11 (4.9)	6 (2.7)
9. Keep up at work or school	1.6 (1.15)	1.47–1.78	112 (50.0)	52 (23.2)	18 (8.0)	16 (7.1)	6 (2.7)
10. Miss work or school	1.3 (0.99)	1.17–1.43	149 (66.5)	26 (11.6)	14 (6.3)	6 (2.7)	5 (2.2)
11. Disturbed sleep	1.9 (1.07)	1.77–2.06	106 (47.3)	58 (25.9)	38 (17.0)	17 (7.6)	5 (2.2)
*Week 24 EoE-IQ score* (*n* = 212)							
1. Bothered by symptoms	2.2 (1.03)	2.09–2.37	51 (24.1)	95 (44.8)	40 (18.9)	18 (8.5)	8 (3.8)
2. Worried about swallowing	2.0 (1.09)	1.90–2.19	80 (37.7)	75 (35.4)	31 (14.6)	19 (9.0)	7 (3.3)
3. Worried about choking	1.8 (1.00)	1.62–1.90	114 (53.8)	55 (25.9)	27 (12.7)	12 (5.7)	4 (1.9)
4. Embarrassed	1.4 (0.84)	1.27–1.50	164 (77.4)	28 (13.2)	11 (5.2)	5 (2.4)	4 (1.9)
5. Worried about swallowing in public	1.8 (1.10)	1.63–1.93	117 (55.2)	54 (25.5)	19 (9.0)	14 (6.6)	8 (3.8)
6. Difficulty in social activities	1.5 (0.94)	1.39–1.65	148 (69.8)	36 (17.0)	12 (5.7)	14 (6.6)	2 (0.9)
7. Family relationships	1.2 (0.59)	1.12–1.28	185 (87.3)	13 (6.1)	13 (6.1)	0 (0.0)	1 (0.5)
8. Friendships	1.2 (0.61)	1.13–1.30	184 (86.8)	15 (7.1)	10 (4.7)	2 (0.9)	1 (0.5)
9. Keep up at work or school	1.2 (0.83)	1.04–1.27	142 (67.0)	27 (12.7)	8 (3.8)	5 (2.4)	1 (0.5)
10. Miss work or school	1.1 (0.75)	0.98–1.18	152 (71.7)	17 (8.0)	10 (4.7)	2 (0.9)	1 (0.5)
11. Disturbed sleep	1.5 (0.80)	1.36–1.58	144 (67.9)	46 (21.7)	14 (6.6)	7 (3.3)	1 (0.5)

CI, confidence interval; EoE-IQ, Eosinophilic Esophagitis Impact Questionnaire; SD, standard deviation

**Table 3:** Descriptive statistics for EoE-IQ average score at baseline and week 24

Statistic	Baseline (N = 239)	Week 24 (N = 227)	Change baseline to week 24 (N = 227)
n	224	212	198
Mean (SD)	2.27 (0.770)	1.57 (0.648)	− 0.66 (0.645)
Median	2.09	1.36	− 0.55
Q1: Q3	1.73: 2.64	1.09: 1.79	− 1.00: − 0.18
Min: max	1.0: 4.9	1.0: 4.7	− 2.8: 1.9
% of patients with worst (highest) EoE-IQ average score (floor effect)	0%	0%	Not applicable
% of patients with best (lowest) EoE-IQ average score (ceiling effect)	0.4%	14.6%	Not applicable
% missing	15 (6.3%)	15 (6.6%)	29 (12.8%)

EoE-IQ, Eosinophilic Esophagitis Impact Questionnaire; Q1, quartile 1; Q3, quartile 3; SD, standard deviation

Inter-item and corrected item-total correlations for the EoE-IQ at baseline are presented in Additional file 1: Table S2. All items showed moderate to strong (|r|≥ 0.30) correlations with several other EoE-IQ items, and all inter-item correlations were below 0.80 (except between Item 7 [Family relationships] and Item 8 [Friendship], r = 0.88), suggesting minimal redundancy. Item 10 (Miss work or school) had a strong correlation with Item 9 (Keep up at work or school; r = 0.70); moderate correlations of 0.31 to 0.42 with Item 1, Item 6 (Difficulty in social activities), Item 7, and Item 8; and low correlations with the emotional functioning items (Item 2, Item 3 [Worried about choking], Item 4 [Embarrassed], and Item 5). In addition, the correlations between each item and the corresponding corrected total score (i.e., the sum of the other EoE-IQ items) were all above 0.50 (range: 0.56 to 0.79), except for Item 10 (r = 0.37). Overall, these results suggest that EoE-IQ items were appropriately related.

### Reliability

Cronbach’s coefficient alphas for the EoE-IQ were 0.89 at baseline and 0.91 at week 24, indicating high internal consistency. Test–retest reliability ICCs for the EoE-IQ average score between week 12 and week 24 were above the minimum value of 0.70 recommended to indicate acceptable reliability [19, 21], for both the subsample of patients with no change in PGIS (ICC = 0.75; n = 109) and the subsample of patients with no change in PGIC (ICC = 0.81; n = 102).

### Construct validity

#### Convergent and divergent validity

Table 4 shows the construct validity correlations (i.e., convergent and divergent validity) between the EoE-IQ average scores and scores on other study measures at baseline and week 24. The evaluated correlations were higher at week 24 than at baseline, and the EoE-IQ positively correlated with all other measures except for the small/weak negative correlation (− 0.10) with EoE-EREFS at baseline. As expected, the correlations between EoE-IQ scores and other EoE-specific PRO scores (i.e., PGIS, DSQ, and EoE-SQ) were moderate at baseline (|r|= 0.44–0.60) and moderate to strong at week 24 (|r|= 0.61–0.72). Correlations with the clinical and histology measures tended to be lower.

**Table 4:** Summary of key measurement properties of the EoE-IQ at baseline and week 24

Measurement property		Baseline	Week 24
*Construct validity: convergent and divergent validity*			
Pearson correlation coefficient (n) at baseline/week 24	DSQ	0.44 (224)	0.68 (175)
	EoE-SQ Frequency	0.56 (224)	0.72 (212)
	EoE-SQ Severity	0.54 (224)	0.61 (212)
	PGIS	0.60 (224)	0.67 (212)
	EoE-EREFS	–0.10 (224)	0.20 (207)
	Peak esophageal intraepithelial eosinophil count	Not conducted^a^	0.15 (208)
	Categorized peak esophageal intraepithelial eosinophil count	Not conducted^a^	0.09 (208)
*Construct validity: known-groups validity*			
Mean score (n) per known group at baseline/week 24	PGIS	None: 1.09 (1)	None: 1.15 (66)
		Mild: 1.82 (99)	Mild: 1.52 (107)
		Moderate: 2.52 (104)	Moderate: 2.29 (32)
		Severe: 3.25 (20)	Severe: 2.99 (7)
		*P* < 0.0001	*P* < 0.0001
	Categorized peak esophageal intraepithelial eosinophil count	≤ 6 eos/hpf: not conducted^a^	≤ 6 eos/hpf: 1.49 (97)
		> 6 to < 15 eos/hpf: not conducted^a^	> 6 to < 15 eos/hpf: 1.54 (30)
		≥ 15 eos/hpf: not conducted^a^	≥ 15 eos/hpf: 1.61 (81)
		*P* = not conducted^a^	*P* = 0.4556
*Ability to detect change: responsiveness and interpretation of change*			
EoE-IQ change by PGIS change between baseline and week 24	Mean change score (n)	–	Improved: –0.863 (121)
		–	No change: − 0.394 (65)
		–	Worsened: 0.062 (12)
		–	*P* < 0.0001
EoE-IQ change by PGIC at week 24	Mean change score (n)	–	Better: − 0.780 (160)
		–	No change: − 0.231 (29)
		–	Worse: 0.251 (9)
		–	*P* < 0.0001
Pearson correlation of change coefficient (n) at week 24	DSQ	–	0.46 (162)
	EoE-SQ Frequency	–	0.53 (198)
	EoE-SQ Severity	–	0.42 (198)
	PGIS	–	0.55 (198)
	PGIC	–	0.44 (198)
	EoE-EREFS	–	0.04 (194)
	Peak esophageal intraepithelial eosinophil count	–	0.07 (195)
	Categorized peak esophageal intraepithelial eosinophil count	–	0.03 (195)

DSQ, Dysphagia Symptom Questionnaire; EoE-EREFS, Eosinophilic Esophagitis Endoscopic Reference Score; EoE-IQ, Eosinophilic Esophagitis Impact Questionnaire; EoE-SQ, Eosinophilic Esophagitis Symptom Questionnaire; eos/hpf, eosinophils per high-power field; PGIC, Patient Global Impression of Change; PGIS, Patient Global Impression of Severity

^a^Analysis not conducted because the study inclusion criteria required patients to have eos/hpf ≥ 15 at baseline, thereby limiting the dynamic range of scores

#### Known-groups validity

Table 4 presents mean EoE-IQ average scores for known groups defined by PGIS categories at baseline and week 24 and peak esophageal intraepithelial eosinophil count at week 24. At both timepoints, higher mean scores were observed with increasingly more severe disease as defined by PGIS, and omnibus tests were statistically significant at baseline and week 24 (*P* < 0.0001). Higher mean EoE-IQ average scores were also observed for patients with higher peak esophageal intraepithelial eosinophil counts at week 24 (i.e., worse histological response); while the trend of EoE-IQ means was as expected, the between-group differences were small and not statistically significant.

### Responsiveness

Table 4 presents mean EoE-IQ average scores for patients who had improved, not changed, and worsened at week 24 based on PGIS and PGIC. The highest negative EoE-IQ change scores (indicating the greatest improvement) were observed for patients who had improved on the global assessments. The omnibus tests were statistically significant for both PGIS and PGIC (*P* < 0.0001). The within-group change effect sizes were large for both PGIS-improved (SES =  − 1.40) and PGIC-improved groups (SES =  − 1.29); the change effect sizes were also large between the response groups (improved/better, no change, worsened/worse) defined by PGIS and PGIC (SES =  − 1.68 to − 0.81) (Additional file 1: Table S3). The pattern of responsiveness correlations was generally consistent with the cross-sectional correlations (Table 4), with EoE-IQ scores showing the largest correlations with change scores on other EoE-specific PRO measures and the smallest correlations with changes in the endoscopic and histologic measures.

### Interpretation of change

The adequacy of the PGIS and PGIC as anchor measures for the EoE-IQ average score was confirmed by the correlations of change from baseline to week 24 (r = 0.55 with the change in PGIS and 0.44 with PGIC). In addition, the mean and median changes in EoE-IQ average scores across the levels of each anchor measure were as expected, with increasing negative scores (representing improvement) generally associated with greater levels of improvement on the PGIS and PGIC (Table 5). Using the anchor of a 1-point improvement on the PGIS from baseline to week 24, the median EoE-IQ change score was − 0.64 (n = 93, mean =  − 0.71, 95% CI =  − 0.83 to − 0.60). Using the PGIC anchor of “A little better” at week 24, the median EoE-IQ change score was − 0.55 (n = 44, mean =  − 0.61, 95% CI =  − 0.79 to − 0.43). Additionally, the lower limit of the 95% CI for patients with “no change” on the PGIS and PGIC was − 0.51 and − 0.37, respectively. The empirical CDF plots showed clear separation of the curves across varying levels of change on the PGIS (Additional file 1: Figures S1, S2), supporting the use of this measure to determine meaningful improvement in EoE-IQ average score.

**Table 5:** Change from baseline in EoE-IQ average score at week 24 using PGIS and PGIC anchor measures

Anchor measure/anchor group	EoE-IQ average change score from baseline to week 24			
	n	Mean (SD)	95% CI	Median
*PGIS*				
Worsening (≥ 1-point worsening)	12	0.062 (0.7182)	− 0.3943 to 0.5183	− 0.091
0-point change	65	− 0.394 (0.4854)	− 0.5146 to − 0.2740	− 0.364
1-point improvement	93	− 0.713 (0.5527)	− 0.8268 to − 0.5992	− 0.636
2-point improvement	25	− 1.277 (0.5060)	− 1.4857 to − 1.0680	− 1.222
3-point improvement	3	− 2.054 (0.6833)	− 3.7513 to − 0.3564	− 1.909
*PGIC*				
Worsening (A little worse, Moderately worse, Very much worse)	9	0.251 (0.7648)	− 0.3365 to 0.8393	0.182
No change	29	− 0.231 (0.3785)	− 0.3747 to − 0.0868	− 0.091
A little better	44	− 0.610 (0.5780)	− 0.7852 to − 0.4338	− 0.545
Moderately better	53	− 0.842 (0.6140)	− 1.0112 to − 0.6727	− 0.700
Very much better	63	− 0.848 (0.6067)	− 1.0005 to − 0.6949	− 0.818

CI, confidence interval; EoE-IQ, Eosinophilic Esophagitis Impact Questionnaire; PGIC, Patient Global Impression of Change; PGIS, Patient Global Impression of Severity; SD, standard deviation

As expected, the distribution-based estimates were lower than the anchor-based estimates; the half-SD of the baseline score was 0.39, and the SEM using reliability based on PGIS was 0.39. Taken together, the anchor-based and distribution-based estimates, especially the median value corresponding to a 1-point improvement on the PGIS, indicated that a minimum 0.6-point reduction in the EoE-IQ average score could be considered a clinically meaningful within-patient change.

## Discussion

The EoE-IQ is a novel instrument for evaluating the impact of EoE in adults and adolescents, and this analysis of the EoE-IQ is, to our knowledge, the first psychometric evaluation of an EoE-specific HRQOL measure using data from a randomized phase 3 trial. While clinical trials in EoE appropriately focus on the symptom of dysphagia as a co-primary endpoint, given the impacts of EoE symptoms on broader aspects of patients’ daily lives and functioning, it is important to understand how new treatment options can alleviate these other impacts as well. Patient-reported outcome measures—such as the EoE-IQ—that are well-defined, reliable, valid, and responsive for use with both adolescents and adults in a clinical trial setting are needed to comprehensively capture patients’ experiences with treatment.

These analyses support the EoE-IQ as a valid and reliable measure to assess the patient-reported impact of EoE among adult and adolescent patients, complementing evidence of the content validity of the measure. The psychometric results revealed that the EoE-IQ average score has adequate distributional properties, measurement structure, internal consistency, test–retest reliability, convergent and divergent construct validity, known-groups validity, and ability to detect change. Notably, the EoE-IQ was shown to correlate as expected with other EoE-specific patient-reported measures (i.e., PGIS, PGIC, EoE-SQ, and DSQ) and did so more closely than with clinical and endoscopic measures (i.e., EoE-EREFS and peak esophageal intraepithelial eosinophil counts). Patterns of change scores and large between-group effect sizes in comparisons between subgroups with and without improvement (as defined by PGIS and PGIC) demonstrated the ability of the EoE-IQ to detect change. In addition, when using a 1-point improvement on the PGIS as the primary anchor to explore thresholds for within-patient change, a 0.6-point reduction in the EoE-IQ average score is proposed as a clinically meaningful improvement on this measure.

While existing measures are available to evaluate HRQOL in patients with EoE, these measures were developed for either an adult population or a pediatric population [12–14]. By evaluating the HRQOL impacts of concern across both adolescents and adults, the EoE-IQ complements validated measures of EoE symptoms, such as the DSQ [16, 17], enabling a comprehensive assessment of patients’ treatment experience. Further, the low levels of association between EoE-IQ scores and the EoE-EREFS and peak esophageal intraepithelial eosinophil count observed in our analysis support findings from previous research that the symptoms and impacts of EoE are not well correlated with histologic or endoscopic endpoints [25, 26]. The discordance observed between patient-reported impacts and histologic or endoscopic endpoints of EoE may be driven by different rates of placebo response in patient-reported versus clinical outcomes [27] or by differences in time to symptomatic versus histologic response [17], thus emphasizing the need for a specific measure to capture HRQOL impacts in EoE.

This analysis is strengthened by the use of data from a phase 3 trial involving both adults and adolescents. Although the analysis sample of participants in the R668-EE-1774 trial was selected according to study eligibility criteria, participants in the trial were representative of the overall EoE patient population [15]. Questionnaire development, which included qualitative input from both adults and adolescents, and the psychometric methods used to evaluate the EoE-IQ were consistent with best practices [11, 28, 29]. Qualitative patient input on meaningful EoE-IQ score change would complement the anchor-based analyses presented here and inform the interpretation of meaningful change in EoE-IQ average score, providing further evidence of the validity of this novel measure.

In summary, the 11-item EoE-IQ focuses on concepts of importance to patients, is easy to administer, and is able to detect clinically meaningful change with treatment. Future validation studies may explore application of the EoE-IQ in contexts of use other than the clinical trial setting.

## Conclusion

The psychometric evaluation of the EoE-IQ using study R668-EE-1774 data confirmed the measure as fit-for-purpose to assess the HRQOL impact of EoE among adolescent and adult patients. The EoE-IQ has acceptable distributional properties, construct validity, reliability, and ability to detect change and can be considered for use in future studies conducted in clinical research settings.

### Supplementary Information

**Additional file 1.** Supplemental Material.

## Data Availability

Qualified researchers may request access to study documents (including the clinical study report, study protocol with any amendments, blank case report form, and statistical analysis plan) that support the methods and findings reported in this manuscript. Individual anonymized participant data will be considered for sharing once the indication has been approved by a regulatory body, if there is legal authority to share the data and there is not a reasonable likelihood of participant re-identification. Submit requests to https://vivli.org/.

## Abbreviations

ANOVA: Analysis of variance
CDF: Cumulative distribution function
CI: Confidence interval
DSQ: Dysphagia Symptom Questionnaire
EoE: Eosinophilic esophagitis
eos/hpf: Eosinophils per high-power field
EoE-EREFS: Eosinophilic Esophagitis Endoscopic Reference Score
EoE-IQ: Eosinophilic Esophagitis Impact Questionnaire
EoE-SQ: Eosinophilic Esophagitis Symptom Questionnaire
HRQOL: Health-related quality of life
ICC: Intraclass correlation coefficient
PGIC: Patient Global Impression of Change
PGIS: Patient Global Impression of Severity
PRO: Patient-reported outcome
SD: Standard deviation
SEM: Standard error of measurement
SES: Standardized effect size
