# Supplementary material for: Validation of the novel Eosinophilic Esophagitis Impact Questionnaire
Source: J Patient Rep Outcomes. 2023 Nov 27;7:120. doi: 10.1186/s41687-023-00654-z (PMC10682344; doi:10.1186/s41687-023-00654-z)
Supplement: Supplementary file 1 — Additional file 1. Supplemental Material. [file 41687_2023_654_MOESM1_ESM.docx]

# Supplemental Material

Supplementary Section 1. Overview of the development and content validation of the EoE-IQ

The methods and results of the research activities that informed the development of the EoE-IQ are summarized below.

**Targeted literature review:** A search strategy was developed to identify peer-reviewed articles presenting information on the patient experience with EoE-related symptoms and impacts. A search strategy was developed to identify articles of interest, consisting of terms related to: (1) EoE (e.g., eosinophilic, allergic, esophagitis, EoE), (2) symptoms and impacts (e.g., symptom, impact, burden, activities of daily living), and (3) qualitative research (e.g., interview, focus group, patient experience). The search was conducted on 17 July 2017 in the MEDLINE, Embase, and PsycINFO databases via the OvidSP platform. The search was limited to peer-reviewed, English-language articles presenting results in human subjects that were published within 10 years of the search being conducted (i.e., July 2007 to July 2017).

Abstracts were reviewed for relevance and articles presenting information on EoE-related symptoms and HRQoL impacts in adult and adolescent patient populations were reviewed. Data pertaining to EoE-related symptom and HRQoL impact concepts identified through review of full-text articles were extracted and summarized.

A total of 15 articles were selected for an in-depth review and data extraction. Among these articles, 9 reported data derived from qualitative interviews with patients or reviews of empirical literature. The remaining 6 articles reported quantitative data derived from survey collection, histological reviews, or clinical trial findings. The majority of articles included a mix of both adult and adolescent or pediatric patients with EoE (n=8 articles), while others exclusively included adult patients with EoE (n=5 articles) or patients <18 years of age (including adolescents; n=4 articles).

A total of 27 HRQoL impact concepts associated with EoE were identified across eight domains in 15 articles: (1) eating behavior, (2) physical functioning, (3) emotional functioning, (4) social functioning, (5) work- and school-related impacts, (6) sleep impacts, (7) emergency room visits, and (8) long-term impacts of disease.

**Advice meetings with therapeutic area experts:** Three expert advice meetings (EAMs) were conducted with clinicians who treat individuals with EoE, specialize in gastroenterology (n=2) or allergy-immunology (n=1), and had at least 12 years’ experience treating EoE. Each EAM lasted approximately 60 minutes and was conducted by two trained researchers by telephone. Discussions were facilitated using open-ended questions to elicit information pertaining to the disease-related symptoms and HRQoL impacts that, from the perspective of the experts, were important and relevant to the experience of adult and adolescent patients with EoE. Probing questions related to the difference in disease experience between age groups (i.e., adults and adolescents) were also included in the interview guide. Experts reported a total of 17 HRQoL impacts across eight domains: (1) eating behavior, (2) emotional functioning, (3) social functioning, (4) work- and school-related impacts, (5) sleep impacts, (6) physical functioning, (7) financial impacts, and (8) impacts relating to medical care. Several impact concepts were reported by all three experts, including modifying eating behavior (eating behavior domain), food elimination or avoidance (eating behavior domain), symptom-triggered anxiety (emotional functioning), and financial impact due to disease management.

**Concept selection, item generation, and questionnaire construction:** The impact-related concepts most frequently reported by experts and identified in the literature were selected for inclusion in the EoE-IQ. Consideration was also given to concepts reported to be most important during the EAMs and the effectiveness of assessing the concept by a subjective PRO rather than an objective clinical assessment (e.g., weight loss). In total, 11 concepts were selected for inclusion across four hypothesized domains: emotional functioning (5 items assessing feelings of bother; worry about trouble swallowing, choking, or swallowing in public; and embarrassment), social impacts (3 items assessing impact on eating-related social activities, relationships with family and friends), school/work impacts (2 items assessing difficulty keeping up with work/school and missing work/school because of EoE) and interrupted sleep due to EoE (1 item). After concepts were selected, an initial version of the EoE-IQ was developed including instructions, item language, response options (i.e., 5-point verbal rating scale ranging from “Not at all” to “Extremely” to assess level of impact), and recall period (i.e., past 7 days).

**Cognitive debriefing interviews:** 23 cognitive debriefing interviews (CDIs) were conducted, with adult and adolescent patients with EoE (>12 years of age), to evaluate participants’ ability to understand the EoE-IQ as intended and confirm the relevance and comprehensiveness of the concepts measured by the questionnaire. The study protocol and related documents received independent review board approval from Copernicus Group Independent Review Board. During 11 of the interviews, the EoE-IQ was debriefed in full with both adult (n=5) and adolescent (n=6) participants. In the remaining 12 interviews, adult participants were asked to read the EoE-IQ and provide feedback (without any follow-up questions being asked). After interviews were conducted, the qualitative data were analyzed.

Results of the 11 CDIs in which the EoE-IQ was debriefed in full indicated that the EoE-IQ was well understood by participants with nine of the 11 EoE-IQ items being interpreted as intended by all 11 participants. Further, findings indicated that the EoE-IQ measured impact concepts relevant to the patient experience with EoE; eight of the 11 EoE-IQ item concepts were reported to be relevant by >60% of the participants (n≥7, 64%). The three remaining concepts were endorsed by more than 1/3 of the sample (n>4, 36%) and as such, no concepts were removed from the draft questionnaire. Further, 10 of the 11 patients interviews (90.9%) reported that no concepts were missing from the questionnaire; the one patient who reported a concept was missing (n=1, 9.1%) suggested the addition of an item that assess the general impact of EoE. As this concept was broad (and would likely be assessed by the EoE-IQ total score), this item was not added to the questionnaire. The response options and recall period for each item of the EoE-IQ were reported to be suitable by >73% of the participants asked.

Findings in the 12 CDIs in which the EoE-IQ was completed but not fully debriefed further supported these findings. No participants spontaneously expressed confusion related to any aspect of the questionnaires and no concepts were reported to be irrelevant to the EoE population as a whole (though a minority of participants noted concepts may not be relevant to their own experience).

The findings of the 23 CDIs conducted with adult and adolescent patients with EoE support the content validity of the EoE-IQ in the intended target patient population as a questionnaire that can be used to assess EoE-related impact concepts that are important and relevant to patients with EoE, in ways that patients can understand.

Supplementary Table 1. Supporting Measures Used in the Analyses

| Outcome Measure, Domains/Items, and Recall | Response Scale and Scoring |
| --- | --- |
| **EoE-related PRO measures** |  |
| **Patient Global Impression of Severity (PGIS)**  Single item questionnaire providing a self-assessment of the severity of difficulty swallowing food  Past week recall | 4‑point response scale (1 = None to 4 = Severe); lower values indicate lower symptom severity |
| **Patient Global Impression of Change (PGIC)**  Single item questionnaire providing a self-assessment of change in difficulty swallowing food since the patient started taking the study medication | 7-point response scale (0 = Very much better to 6 = Very much worse); lower values indicate greater improvement in difficulty swallowing food since the start of treatment |
| **Eosinophilic Esophagitis Symptom Questionnaire (EoE-SQ)**  Measures the frequency and severity of 5 EoE symptoms:   - Chest pain - Stomach pain - Burning feeling in the chest - Food or liquid coming back up into the throat - Throwing up   Past 7 days recall | EoE-SQ Frequency:   - 5-point scale (1 = Never to 5 = More than once a day) - Score is calculated as the sum of frequency scores from 5 items (range, 5 to 25); higher score indicates greater frequency   EoE-SQ Severity   - 11-point numeric rating scale (0 = No symptom to 10 = Worst possible symptom) - Score is calculated as the sum of severity scores from 3 items (chest pain, stomach pain, and burning feeling in the chest) (range, 0 to 30); higher scores indicate greater severity |
| **Dysphagia Symptom Questionnaire (DSQ) [16,17]**  Assesses daily frequency and severity of dysphagia associated with EoE in adults and adolescents   - Item 1 asks whether the patient ate solid food - If yes, Item 2 assesses the frequency of dysphagia and Item 3 assesses the severity of dysphagia - Item 4 assesses pain with swallowing food   Daily recall | - Daily scores are derived based on responses to Items 2 and 3 (range, 0 to 6) - DSQ biweekly total score is the sum of daily scores through 14 days (range, 0 to 84); higher scores indicate more severe dysphagia |
| **Clinical measures** |  |
| **Peak esophageal intraepithelial eosinophil count**  Maximum quantity of eosinophils in the most inflamed high-power fields (eos/hpf) across 3 regions of the esophagus | Can be categorized into 3 levels:   - ≤6 (histological remission) - >6 to <15 - ≥15 eos/hpf (threshold for EoE diagnosis/inclusion in R668-EE-1774) |
| **Eosinophilic Esophagitis Endoscopic Reference Score (EoE-EREFS) [18]**  System for scoring inflammatory and remodeling features of EoE for the proximal and distal esophageal regions | Total score (summing scores for the 2 regions) ranges from 0 to 18, with higher scores indicating greater disease activity |

EoE = Eosinophilic Esophagitis; PRO = patient-reported outcome.

Supplementary Table 2. Correlations of EoE-IQ Item-Item and Item-Total Score at Baseline

|  | **Correlation coefficient** | | | | | | | | | | |
| --- | --- | --- | --- | --- | --- | --- | --- | --- | --- | --- | --- |
| **EoE-IQ Items** | **1.** | **2.** | **3.** | **4.** | **5.** | **6.** | **7.** | **8.** | **9.** | **10.** | **11.** |
| n | 224 | 224 | 224 | 224 | 224 | 224 | 224 | 224 | 204 | 200 | 224 |
| 1. Bothered by symptoms | ─ |  |  |  |  |  |  |  |  |  |  |
| 2. Worried about swallowing | 0.64 | ─ |  |  |  |  |  |  |  |  |  |
| 3. Worried about choking | 0.52 | 0.75 | ─ |  |  |  |  |  |  |  |  |
| 4. Embarrassed | 0.49 | 0.50 | 0.54 | ─ |  |  |  |  |  |  |  |
| 5. Worried about swallowing in public | 0.44 | 0.65 | 0.68 | 0.66 | ─ |  |  |  |  |  |  |
| 6. Difficulty in social activities | 0.58 | 0.60 | 0.57 | 0.57 | 0.65 | ─ |  |  |  |  |  |
| 7. Family relationships | 0.45 | 0.45 | 0.47 | 0.57 | 0.40 | 0.66 | ─ |  |  |  |  |
| 8. Friendships | 0.44 | 0.44 | 0.42 | 0.53 | 0.42 | 0.73 | 0.88 | ─ |  |  |  |
| 9. Keep up at work or school | 0.54 | 0.49 | 0.46 | 0.47 | 0.34 | 0.63 | 0.70 | 0.74 | ─ |  |  |
| 10. Miss work or school | 0.33 | 0.12 | 0.13 | 0.17 | 0.05 | 0.31 | 0.38 | 0.42 | 0.70 | ─ |  |
| 11. Disturbed sleep | 0.45 | 0.35 | 0.37 | 0.38 | 0.23 | 0.50 | 0.60 | 0.61 | 0.64 | 0.49 | ─ |
| Corrected total score^a^ | 0.66 | 0.68 | 0.67 | 0.64 | 0.62 | 0.79 | 0.70 | 0.72 | 0.71 | 0.37 | 0.56 |

EoE-IQ, Eosinophilic Esophagitis Impact Questionnaire.

^a^ The corrected total score is computed without the corresponding item.

For EoE-IQ, polychoric correlations are calculated between item scores, and polyserial correlations are calculated between these items and the corresponding corrected total scores.

Supplementary Table 3. Responsiveness Effect Sizes for Interpretation of Change in the EoE-IQ

| Statistic | Estimate |
| --- | --- |
|  |  |
| **Responsiveness: Effect Sizes** |  |
| **EoE-IQ change by PGIS change between baseline and week 24** |  |
| Within-group SES |  |
| Improved | –1.40 |
| No change | –0.81 |
| Worsened | 0.09 |
| Between-group SES |  |
| Improved vs. no change | –0.81 |
| Improved vs. worsened | –1.48 |
| No change vs. worsened | –0.87 |
| **EoE-IQ change by PGIC at week 24** |  |
| Within-group SES |  |
| Better | –1.29 |
| No change | –0.61 |
| Worse | 0.33 |
| Between-group SES |  |
| Better vs. no change | –0.95 |
| Better vs. worse | –1.68 |
| No change vs. worse | –0.98 |

PGIC, Patient Global Impression of Change; PGIS, Patient Global Impression of Severity; SD, standard deviation; SEM, standard error of measurement; SES, standardized effect size.

Supplementary Figure 1. Empirical Cumulative Distribution Function of Change from Baseline in EoE-IQ Average Score to Week 24, by Change in PGIS Score (5-Level Categorization) at Week 24

EoE-IQ, Eosinophilic Esophagitis Impact Questionnaire; PGIS, Patient Global Impression of Severity.

Empirical cumulative distribution function plots provide visual support for the estimation of within-patient change thresholds. Less overlap in the curves is preferred and indicates greater differentiation between groups experiencing change on the anchor measure.

Supplementary Figure 2. Empirical Cumulative Distribution Function of Change from Baseline in EoE-IQ Average Score to Week 24, by PGIC Score (5-Level Categorization) at Week 24

EoE-IQ, Eosinophilic Esophagitis Impact Questionnaire; PGIC, Patient Global Impression of Change.

Empirical cumulative distribution function plots provide visual support for the estimation of within-patient change thresholds. Less overlap in the curves is preferred and indicates greater differentiation between groups experiencing change on the anchor measure.
